# Overhauling the ecotoxicological impact of synthetic pesticides using plants’ natural products: a focus on *Zanthoxylum* metabolites

**DOI:** 10.1007/s11356-023-27258-w

**Published:** 2023-05-06

**Authors:** Innocent Uzochukwu Okagu, Emmanuel Sunday Okeke, Wisdom Chinedu Favour Ezeorba, Joseph Chinedum Ndefo, Timothy Prince Chidike Ezeorba

**Affiliations:** 1 Department of Biochemistry, Faculty of Biological Sciences, University of Nigeria, Nsukka, Enugu State, 410001 Nigeria; 2 Natural Science Unit, School of General Studies, University of Nigeria, Nsukka, Enugu State, 410001 Nigeria; 3 Institute of Environmental Health and Ecological Security, School of Environment and Safety Engineering, Jiangsu University, Zhenjiang, 212013 Jiangsu China; 4 Department of Chemistry Education, Faculty of Sciences, Ekiti State University, Ado Ekiti, Ekiti Nigeria; 5 Department of Science Laboratory Technology, University of Nigeria, Nsukka, Enugu State, 410001 Nigeria; 6 Department of Genetics and Biotechnology, Faculty of Biological Sciences, University of Nigeria, Nsukka, Enugu State, 410001 Nigeria; 7 Department of Molecular Biotechnology, School of Biosciences, University of Birmingham Edgbaston, Birmingham, B15 2TT, United Kingdom

**Keywords:** Synthetic pesticides, Herbicides, Insecticides, Fungicide, *Zanthoxylum species*, Ecotoxicology, Health effects

## Abstract

The reduction in agricultural production due to the negative impact of insects and weeds, as well as the health and economic burden associated with vector-borne diseases, has promoted the wide use of chemicals that control these “enemies.” However, the use of these synthetic chemicals has been recognized to elicit negative impacts on the environment as well as the health and wellbeing of man. In this study, we presented an overview of recent updates on the environmental and health impacts of synthetic pesticides against agro-pest and disease vectors while exhaustive reviewing the potentials of natural plant products from *Zanthoxylum species* (*Rutaceae*) as sustainable alternatives. This study is expected to spur further research on exploiting these plants and their chemicals as safe and effective pesticide entities to minimize the impact of their chemical and synthetic counterparts on health and the environment.

**Graphical abstract:**

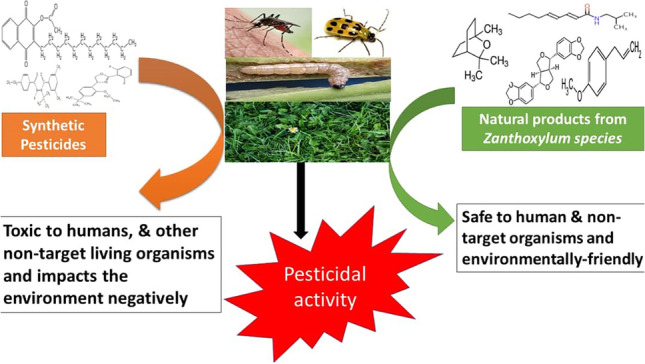

## Introduction

The growing trends in urbanization and industrialization in the past few years, with a corresponding ever-growing population, have greatly affected the ecosystem. This global population increase has cast tremendous pressure on existing agricultural practices. Most countries aim to increase food production to meet their growing population demands, predicted to reach nearly 10 billion by 2050 (Yadav et al. 2020). Different agrochemicals, such as pesticides to combat pests and diseases, have recently been very popular. However, it is necessary to ascertain any adopted agricultural practice’s sustainability and its tantamount effects on human health and the environment. With the increased crop production, natural products must be developed and utilized as sustainable alternatives to synthetic herbicides, insecticides, and other pesticides (Anaduaka et al. 2023).

Synthetic insecticides and herbicides fall under the general class of chemical pesticides. While insecticide specifically kills disease vectors or insects that infest cultivated plants, herbicide kills weeds and unwanted plants competing with cultivated crops for nutrients, sunlight, and water. Insecticides and herbicides can be specific to human enemies or exhibit a broad-spectrum nature, annihilating harmful and beneficial insects and shrub plants (Smith et al. 2021) (Fig. 3). The mode of action differs from one insecticide or herbicide to another. Some insecticides are classified as stomach poison—which elicits their actions upon ingestion by the insect. In contrast, others are contact poisons or fumigants. Contact poisons elicit their action when in contact with the external surface of insects, while fumigants kill insects when inhaled (Campos et al. 2019).

Similarly, most weeds have narrow leaves, which exhibit a wide range of contrasting biochemical properties to their broadleaf counterparts, which are most-time our cultivated shrubs or plants. These biochemical differences form the basis for many herbicides’ selective killing of weeds (Wang et al. 2018). The Weed Science Society of America (WSSA) summarized the biochemical mode of action (MOAs) of generally known herbicides in their publication on http://www.wssa.net/Weeds/Resistance/WSSA-Mechanism-of-Action.pdf. Many known herbicides work with the inhibition of one of the following enzymes or biochemical pathways; acetyl CoA carboxylase (ACCase), acetolactate synthase (ALS), photosystem I and II, fatty acid and lipid biosynthesis, enolpyruvyl shikimate-3-phosphate synthase (EPSPS), glutamine synthetase, carotenoid biosynthesis, protoporphyrinogen oxidase, and mitosis and meiosis (Westwood et al. 2018). Hence, farmers must adopt a suitable herbicide based on their MOAs that best selectively kills weeds without harming the valuable plants. The toxicity of synthetic pesticides and their metabolite on the ecosystem and human health have raised serious global concerns (Wojciechowska et al. 2016).

Alternative pest and weed management methods are the physical and biological methods (Baker et al. 2020). The physical method refers to using physical means to kill or deter pests, insects, rodents, and weeds from adversely affecting humans and their activities. Traps, predators, fires, temperature control, and barriers are the standard physical pest control methods (Vincent et al. 2009). Physical methods generally have minimal adverse health effects, although many results in serious environmental concerns. For instance, burning weeds fosters global warming, and killing birds and rodents may distort ecological balance and favor species extinction (Gerhards and Schappert 2020).

A viable alternative is the adoption of biological controls, which implies using living organisms to control human enemies. Specifically, introducing a predator, parasite, or causative disease agent to a pest may eliminate or distort the viability of that pest without compromising human health and the environment (Stenberg et al. 2021). Recent studies have discovered that plants such as the *Zanthoxylum species* exudate non-human toxic and environmentally-friendly metabolites, potent toxicants to insect pests and weeds (Hikal et al. 2017). This review presents an overview of the health and environmental implications of chemical pesticides while exposing metabolites of *Zanthoxylum species* as a recommended alternative source of natural insecticides and herbicides*.*

## Methodology

This study comprises two parts—the first part (Sections 3 and 4) provides an overview of the ecological and health impacts/consequences of adopting synthetic and chemical pesticides to control agricultural pests and disease vectors. The second part (Section 5) presented an exhaustive review of studies adopting or discovering different natural products or metabolites from *Zanthoxylum species* as pesticides (insecticides, fungicides, and herbicides). This study adopted the classical or traditional review pattern for literature search, using the following keywords—herbicide, insecticides, non-target species, birds, amphibians, fishes, soil ecosystem, agroecosystem, aquatic ecosystem, atmosphere, air, acute toxicity, chronic toxicity, ocular, nasal, dermal, and oral route. Using Boolean connectors such as “AND” or “OR” where necessary, studies specific to the different subsections were retrieved, emphasizing recent studies (2015 – 2022).

In the second section on *Zanthoxylum’s* metabolites, this study adopted the systematic review pattern for retrieving relevant studies. Works of literature reviewed in this section were retrieved from PubMed, Scopus, and ScienceDirect. Moreover, we searched Google Scholar as a secondary source, using essential keywords and Boolean connectors. After synthesizing the retrieved documents from the primary databases, only 81 non-redundant documents were obtained. The systematic search performed on those primary databases adopted keywords such as (*Zanthoxylum*) AND [(herbicide) OR (fungicide) OR (insecticide) OR (pesticides)] in their title, abstract, and keywords. We excluded all non-*Zanthoxylum* literature, works of literature on *Zanthoxylum* as antibiotics, non-English language literature, and literature not specific to pesticide or vector-killing activities. Moreover, our inclusion criteria comprise all studies on products majorly from *Zanthoxylum*, such as extracts, characterized compounds, and grounded plant parts, showing pesticide activities. After applying our exclusion and inclusion criteria, 29 studies were reviewed in the second part of this paper.

## Ecotoxicological impacts of synthetic pesticides

Synthetic pesticides have been reported to impact the environment negatively, affecting the food web and ecosystems in diverse ways. This section discussed the ecotoxicological and health impact of adopting these synthetic pesticides in agriculture pests and disease vector control.

### Impact on non-target species

Despite the ever-growing need and beneficial roles of pesticides, their adverse effect on non-target organisms has been a global concern for decades. Predators act as a biological control measure in controlling the population of pests that are essentially their prey, making them beneficial to the environment. A growing body of evidence from available literature documents the adverse effects of pesticides on the predator population. A plot treated with chlorpyrifos caused a significant reduction in the population of spiders, richness, evenness, and species diversity of collembola relative to the control in a study conducted on grassland in the UK (Fountain et al. 2007; Sánchez-Bayo 2021). Furthermore, foliar application of systemic pesticides of neonicotinoids family such as clothianidin, imidacloprid, admire, acetamiprid, and thiamethoxam resulted in a highly toxic effect on natural enemies when compared to fipronil, buprofezin, and spirotetramat (Kumar et al. 2012; Zhang et al. 2016). Similarly, the spraying of imidacloprid and cypermethrin on the brinjal (also known as nightshades) ecosystem resulted in increased mortality of braconids, predator spiders, and coccinellids when compared to biopesticides and *Azadirachta indica* (neem) as insecticides (Ghananand T, Prasad CS 2011; Zaller and Brühl 2019).

Furthermore, it has been shown that pesticides can cause a change in predator behavior as well as other developmental parameters such as growth rate, development, and reproductive parameters, for instance, a significant decrease in body size, reduction in morphometric parameters, and hemocyte count in *Pterostichus melas italics* (carabid beetle) (Giglio et al. 2011; Benítez et al. 2018). Similarly, glyphosate-based herbicides caused a change in behavior and survival rate of ground beetles and spiders as well as the effect on the arthropod community, thereby influencing ecosystem biological control (Evans et al. 2010; Schmidt-Jeffris et al. 2022).

Pollinators play vital roles in the agricultural process during pollination. Various species of bees (including *Bombus spp*. and *Apis spp*.) and birds and beetles are essential pollinators and serve as bioindicators in the ecosystem. Continuous and indiscriminate use of synthetic insecticides and pesticides could result in low yield/loss of crops by reducing the population of pollinators (Fishel 2014; Ara, 2021). Similarly, other activities of pollinators are disrupted by pesticide (an insecticide) application, ranging from the efficiency of pollen collection, colony mortality, and foraging behavior (Straub et al. 2022).

Neonicotinoid-based insecticides (such as dinotefuran, thiamethoxam, thiacloprid, and clothianidin) caused lethal and sub-lethal effects on learning, foraging behavior, and memory of the bees (Blacquière et al. 2012; Buszewski et al. 2019). Furthermore, exposure of honey bees to a non-lethal dose of thiamethoxam resulted in substantial mortality due to homing failure, thus putting the colony in danger of collapse (Henry et al. 2012; Giri et al. 2018). Case of high mortality, poor efficiency in pollen collection, and colony collapse as a result of exposure of worker bees to pyrethroid and neonicotinoid insecticides has also been reported (Gill et al. 2012; Wood and Goulson 2017). Several recent review articles report other studies on the effects of pesticides on pollinators (Sponsler et al. 2019; Serrão et al. 2022; Straub et al. 2022). In conclusion, synthetic pesticides harm functional and ecologically beneficial non-target species such as pollinators, biological recalcitrant waste degraders, and bees.

### Impact on the soil ecosystem

**Fig. 1:**
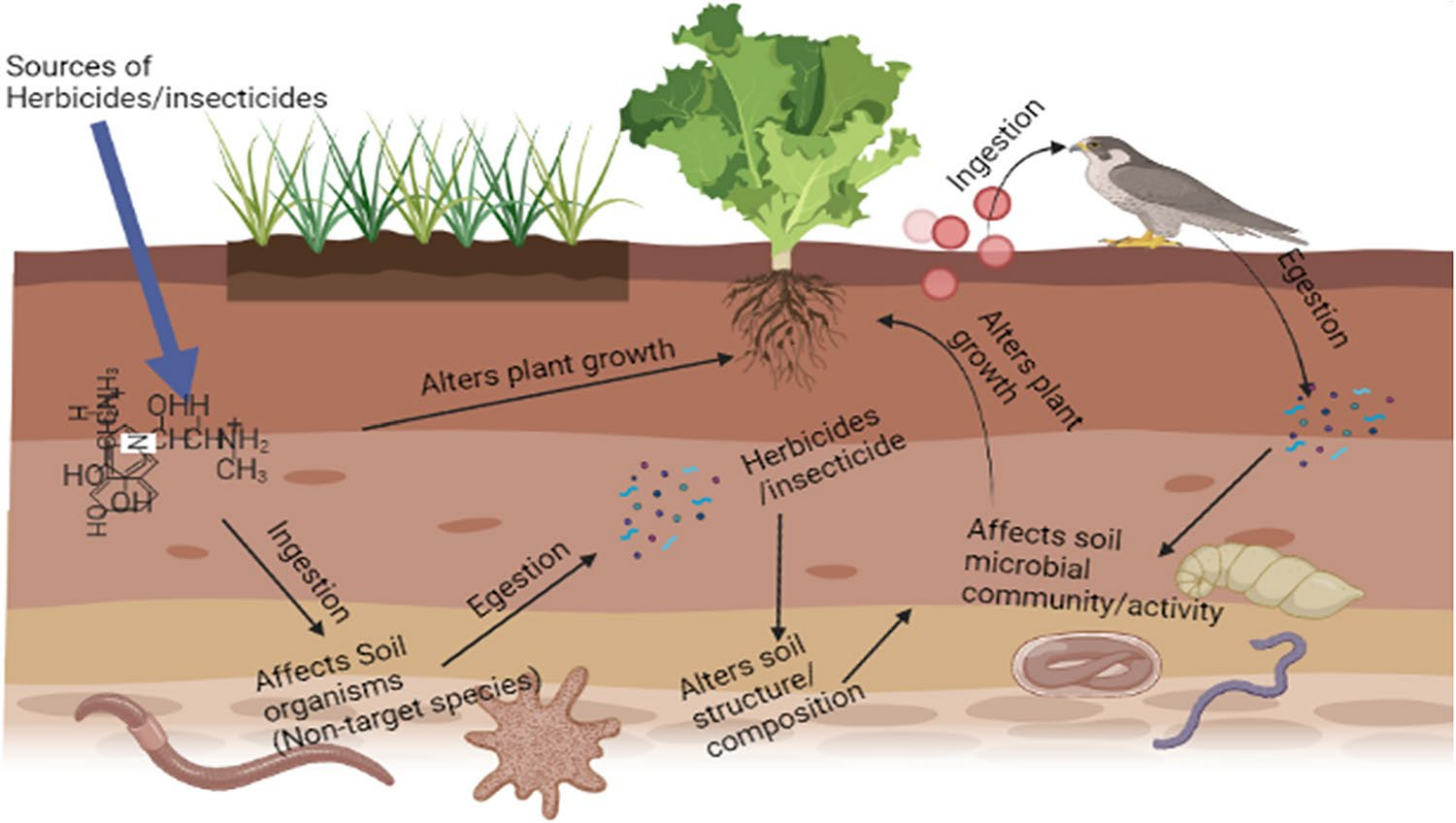
Impact of herbicide/insecticides on soil ecosystem

The continuous use of pesticides in agriculture results in high retention and accumulation of considerable fractions in the soil. The fate of these pesticides is determined by microflora and the properties of the soil, where they undergo various processes such as transport, degradation, adsorption, and desorption (Rasool et al. 2022; Okeke et al. 2022a). After degradation, there is excellent interaction between the pesticides and the soil environment and soil microorganisms, leading to alteration in enzymatic activities, biochemical reactions, and microbial activities (Ahmed and Al-Mutairi 2022; Okeke et al. 2022b). Adversely, certain microbes utilize applied pesticides as an energy source to support their population growth and concomitant soil ecosystem disturbance (Fig. 1). Specifically, chlorpyrifos was found to act as a carbon source, increasing the growth of a bacterial isolate from agricultural soil irrigated with wastewater (Farhan et al. 2021). Several biochemical reactions in the soil, such as ammonification, nitrification, and nitrogen fixation, are adversely affected by herbicides and insecticides via the deactivation/activation of specific soil enzymes and impacting the efficiency and population of soil microorganisms. Whether soil biochemical reaction will be increased or decreased is a function of the synergistic interaction between microorganisms, pesticides, and soil properties (Alengebawy et al. 2021). Within a pesticide concentration of 2.5 kg/ha–5.0 kg/ha, the population of *Azospirillum spp.* was found to be significantly increased, as well as its ammonification rate in vertisol and laterite soils on which *Arachis hypogaea* L. was planted but showed reverse effect at higher concentrations (Srinivasulu et al. 2012).

The rhizosphere microbial community improves soil quality by participating in nutrient and biogeochemical cycles, improving crop yield (Li et al. 2022a). A recent study shows that glyphosate caused a perturbation in the rhizosphere microbial community (Newman et al. 2016; Lu et al. 2017). More so, pesticide applications have also been reported to kill or inhibit some microorganisms and outnumber another group, thereby affecting competition among them. Notably, endosulfan application resulted in a 76% increase in bacterial biomass and a 47% reduction in fungal biomass (Xie et al. 2011; Arora and Sahni 2016). Mineralization of soil organic matter has also been reported to be significantly affected by pesticide application. This crucial soil property influences productivity and soil quality. For instance, soil organic matter was significantly reduced, followed by applying four different herbicides (prime extra, atrazine, glyphosate, and paraquat) (Sebiomo et al. 2011; Athiappan et al. 2022).

Synthetic pesticides have been reported to adversely affect microbial biomass growth, metabolic activities, and colonization (Mandl et al. 2018; Zaller et al. 2018). Also, their negative impacts on valuable soil enzymes, essential in agriculture, decomposition of organic matter, and nutrient cycling cannot be overemphasized (Pattanayak et al. 2022). Other recent findings on the effects of synthetic pesticides on soil and agroecosystems are summarized in recent reviews (Galhardi et al. 2021; Mehdizadeh et al. 2021). We conclude that synthetic pesticides affect the soil and agroecosystems by distorting regular nutrient cycling, soil biomass, mineralization, and agro-productivity.

### Impact on aquatic and air ecosystems

**Fig. 2:**
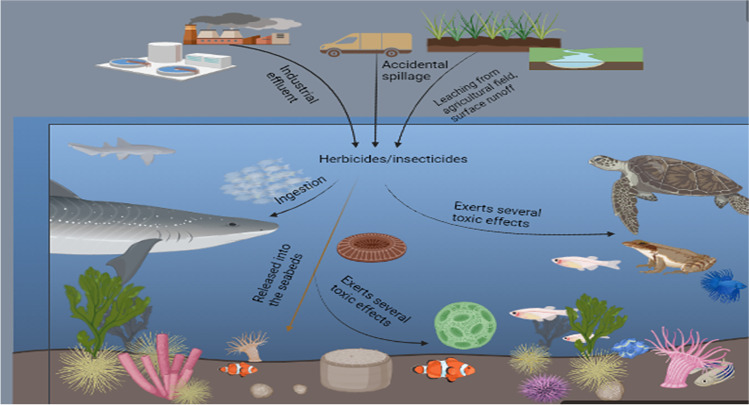
Impact of herbicide/insecticides in aquatic ecosystem

There is uncontrollable downward movement or leaching of pesticides from agricultural fields into water bodies where non-target species, including fishes and other aquatic organisms, are affected, thereby causing a threat to biodiversity and an imbalance in ecosystem equilibrium (Ahmad Dar 2016) (Fig. 2). Pesticides can find their way into the aquatic environment through various routes such as industrial effluent, accidental spillage, washing of spray equipment after spraying, surface runoff, and movement from pesticides-treated agricultural fields (Dhawan et al. 2017). The bioaccumulation of pesticides in the tissues and organs of aquatic organisms has also been reported (Yadav et al., 2018). The continuous deposition of pesticides in the aquatic environment has erupted enormous public health concerns. They pose a significant risk to the aquatic ecosystem and consequent long-term effects on humans throughout the food chain (Kumar et al. 2021).

**Fig. 3:**
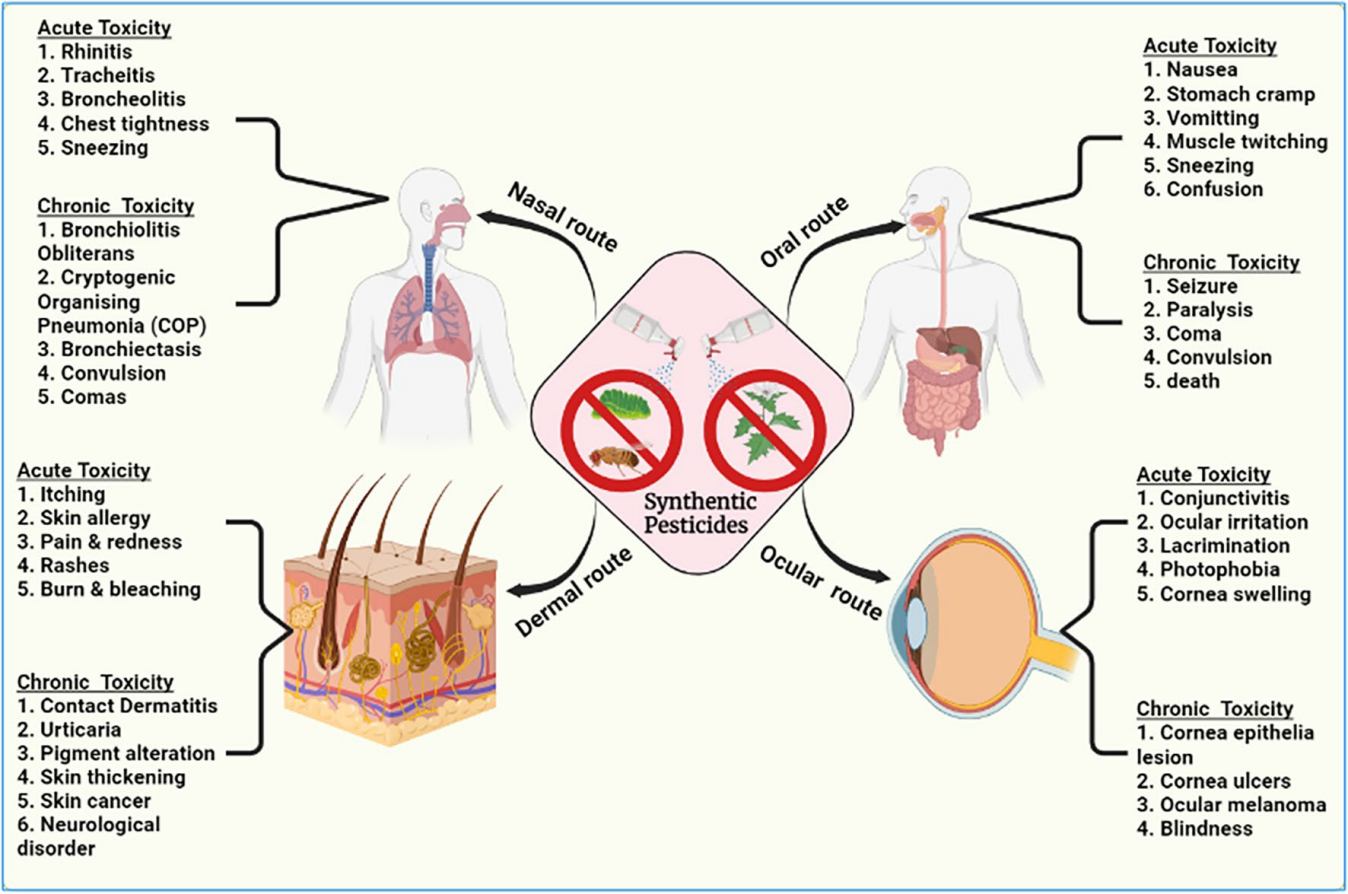
Human health impacts of synthetic pesticides

Similar to the aquatic environment, pesticides in the air can arise through volatilization from applied agricultural fields or surfaces, aerial application, and spray drift. Wind speed and droplet size are key factors determining the drift’s extent. In contrast, the volatilization rate is determined by ambient temperature, wind speed, humidity, the vapor pressure of the pesticide constituents, the surfaces where the pesticides settle, and the time after pesticide treatment (Kaur and Garg 2014). The volatile nature of pesticides poses a significant risk to the atmosphere, making them prone to pollution (Rajmohan et al. 2020). In a study conducted in Washington and California in the United States, organophosphorus pesticides were detected from environmental samples from surfaces and air after spraying agricultural fields (Armstrong et al. 2013; Sharma et al. 2019; Okeke et al. 2022d).

Amphibians are found in various terrestrial, arboreal, and aquatic ecosystems. There are increasing global environmental concerns over the declining population of amphibians worldwide; while most are almost to extinction, a considerable number are already endangered species (Ockleford et al. 2018). Although numerous problems are responsible for such population drop, herbicides and insecticides are significant contributors. Climate change and global warming have caused temperatures to become warmer, thereby increasing the impact of pesticides on the population of amphibians (Johnson et al. 2013; Prasad and Prasad 2022). Other factors include the permeable nature of the skin, the rudimentary immune system, and the terrestrial-aquatic cycle of amphibians (Varga et al. 2018).

Birds are not spared from the hazardous effects of pesticides; they are exposed to these toxic chemicals through ingestion of treated seeds, the spray, treated crops, contaminated water, and contaminated baits (Richard et al. 2021). Certain insecticides, such as organophosphates and carbamates, have been shown to cause bird mortality (Jayaraj et al., 2016). Ground-nesting birds, such as grassland birds in America, were reduced in population due to pesticide toxicity (Mineau and Whiteside, 2013; Gunstone et al. 2021). Various insecticides (such as carbofuran, cholinesterase inhibitors-fenthion, phorate, and carbofuran) and herbicides applied to rice fields have been reported to be highly toxic to birds causing a wide range of mortality and reproductive toxicity (Mladenović et al. 2018) Mladenović, Milan 2018.

The synergistic interaction of fish with the chemical, biological, and physical environment makes them a vital component of the aquatic ecosystem. They are an essential part of the marine food web as they are a food source for other ecosystem members like marine mammals and sea birds. Pesticides have generally been reported to affect fish populations in the aquatic ecosystem, as they have been reported to contribute to the mortality of fish globally. The presence of pesticides in the aquatic environment, even at low concentrations, could still pose a harmful effect on marine organisms (Mladenović et al. 2018). The ability of fishes to bioaccumulate toxic chemicals makes them susceptible to pesticides. Alterations in the biochemical parameters of aquatic organisms could serve as a bioindicator for evaluating the health of aquatic organisms (Poopal et al. 2017). A recent study reported a change in biochemical and histological parameters of *Cirrhinus mrigala* (freshwater fish) after exposure to certain pesticides (Ghayyur et al. 2021). Exposure to pesticides caused severe damage to various fish organs such as the alimentary canal, brain, liver, and gill, suggesting that such concentration of pesticide could cause harm to consumers (Nwani et al. 2021). Another independent study shows that organophosphate pesticides could cause an alteration to vitellogenesis in catfish, thereby hindering catfish farming (Shahjahan et al. 2017). Other toxic effects of pesticides on fishes are the distortion of their olfactory senses, which aids them in locating food, avoiding predators, and mating. Some of these effects and many more are summary in a few recent reviews (Kasumyan 2019). We conclude by asserting from abovementioned studies that synthetic pesticides to greater extent detrimentally affect the aquatic ecosystem and a lesser extent the air ecosystem.

## Impacts of synthetic pesticides on human health

Regardless of the ease of eliminating weeds and insects with herbicides and insecticides, there are countless reports on their adverse effects on human health (Nicolopoulou-Stamati et al. 2016). The predisposing factors that determine the severity of the harmful effects caused by exposure to these toxicants are the nature of the chemical toxicant, length of time of exposure, the quantity taken by the cells, age of the individual, immune status, and presence of underlying ailment or other comorbidities (Alengebawy et al. 2021). These chemicals, on exposure, gain entry through the skin (dermal contact), inhalation into the lungs, orally ingested from the mouth, or making contact with the eyes. In the human or animal body, these herbicides or insecticides could be metabolized, excreted, or bioaccumulated in the body’s adipose (Thapa et al. 2021).

The health implications of insecticides and herbicides range from acute to chronic conditions depending on the severity of the impact (Hassaan and El Nemr 2020) (see Tables 1 and 2). Acute health conditions are short-term and are experienced immediately after a single exposure, whereas chronic conditions are long-term and occur many months and years after exposure. Acute effects or symptoms are immediately felt or experienced on these organs through their entry point. Acute effects include rashes, blisters, coughing, nausea, dizziness, diarrhea, stinging eyes, and even death from extreme toxicity or extended exposure to acute toxicants (Nicolopoulou-Stamati et al. 2016). Chronic conditions are primarily due to the accumulation of toxicants that may seem harmless in the body’s cells over a long period. Chronic conditions are debilitating and range from cancers, tumors, infertility, and reproductive problems to damage to the liver, kidneys, lungs, and other body organs. Summarily, synthetic chemical herbicides and pesticides may cause dermatological, gastrointestinal, cognitive impairment and neurodegeneration, carcinogenic, respiratory, reproductive, and hormonal/endocrine imbalances (Hu et al. 2015; Sarailoo et al. 2022). The health impact of synthetic insecticides and herbicides varies among the different types and classes of the pesticide. The U.S. Environmental Protection Agency (EPA) has classified pesticides based on their toxicity into four categories (I-IV): oral LD_50_, inhalation LC_50_, dermal LD_50_, time-span eye effects, and time-span skin effects. Category I pesticides are regarded as the most toxic pesticide, while category IV is the least toxic (EPA 2014).

**Table 1:** Globally selected insecticides, their mode of action, and adverse health implications

Insecticide class	Active ingredient	Mode of actions	U.S. EPA toxicity category	Acute and chronic health impact	References
Organophosphate	Chlorpyrifos	Inhibition of acetylcholinesterase activity	II	Headache, migraine, sialorrhea and drooling, myoclonus, vomiting, diarrhea, respiratory depression, convulsion, syncope, pinpoint pupils, and endocrine disruptor.	(Darwiche et al. 2018; Ubaid ur Rahman et al. 2021)
	Malathion	Inhibiting acetylcholinesterase and causing cholinergic crises and death in insects like lice.	III	Headache, hypersalivation, muscular dystrophy, nausea, diarrhea, respiratory depression, convulsion, loss of coordination, and anisocoria.	(Severcan et al. 2019; Kata 2020; Badr 2020)
	Methyl parathion	Inhibiting acetylcholinesterase and causing cholinergic crises and death in insects	I	Headache, migraine, sialorrhea and drooling, myoclonus, vomiting, diarrhea, respiratory depression, convulsion, syncope, pinpoint pupils, and endocrine disruption.	(Garcia et al. 2003; Ruckart et al. 2004)
	Phosmet	Inhibiting pseudocholinesterase and acetylcholinesterase and causing cholinergic crises and death in insects	II	Headache, migraine, sialorrhea and drooling, myoclonus, vomiting, diarrhea, respiratory depression, convulsion, syncope, pinpoint pupils, and endocrine disruption.	(Good et al. 1993; Anastassiadou et al. 2021)
	Acephate	By contact or ingestion by the sucking or biting insect, acephate fosters their killing by inhibiting acetylcholinesterase. Hence, acetylcholine accumulated, causing the debilitating cholinergic crisis.	III	Migraine, sialorrhea and drooling, myoclonus, vomiting, diarrhea. Respiratory depression, convulsion, syncope, pinpoint pupils, endocrine disruptor	(Dhanushka and Peiris 2017)
Organochlorine	Endosulfan	Induces ROS-mediated DNA damage in insects’ cells, thereby stimulating DNA damage response (DDR).	I	Allergy, burning, and tingling of the skin, headache, dizziness, lack of coordination, stomach upset, diarrhea, vomiting, tremor, convulsion, nervous coordination loss, hypoventilation, and coma.	(Kucuker et al. 2009; Beyger et al. 2012; Dai et al. 2014; Téllez-Bañuelos et al. 2019)
N-methyl carbamate	Aldicarb	By contact or ingestion by the insects, aldicarb causes cholinergic crises in the insect by inhibiting cholinesterase.	I	Muscle dystrophy, dizziness, profuse perspiration, headache, drooling, vomiting, abdominal cramp, stooling, loss of nervous coordination, and pulmonary edema in severe cases.	(Risher et al. 1987; Baron 1994)
	Carbaryl	Carbaryl reversibly inhibits acetylcholinesterase, causing cholinergic crises, then the death of the insects.	III	Muscle dystrophy, dizziness, profuse perspiration, headache, drooling, vomiting, abdominal cramp, stooling, loss of nervous coordination, and pulmonary edema in severe cases.	(Marian et al. 1983; Fattahi et al. 2012; Toumi et al. 2016; De Jesús Andino et al. 2017; Yang et al. 2020)
Natural origin (flowers of the chrysanthemum family)	Pyrethrins	Naturally contain six refined natural esters from the flower extract that alters the activities of the Na ion channels of the nerves, resulting in nerve firing and death.	III and IV	Skin and upper respiratory tract allergy, asthma, breathing difficulty	(Petroski and Stanley 2009)
Synthetic pyrethrin	Pyrethroids	Contains single synthetic esters that alter the activities of the Na ion channels of the nerves, resulting in nerve firing and death.	II	Facial burn, burning, and tingling of the skin, headache, dizziness, lack of coordination, irritability to cracky noise or touch, diarrhea, vomiting, tremor, convulsion, nervous coordination loss, and numbness.	(Bradberry et al. 2005; Chrustek et al. 2018; Salako et al. 2020)

**Table 2:** Globally selected herbicides, their mode of action, and adverse health implications

Herbicide class	Active ingredients	Mechanism of actions	U.S. EPA toxicity category	Health implication		References
				Acute	Chronic	
Chlorophenoxy herbicides	2,4-Dichloropheno xyacetic acid	An auxin analog that stimulates uncontrollable and lethal growth in plants.	III	Skin allergy, nausea, headache, diarrhea, confusion, and mode change. Muscle weakness in occupationally exposed individuals	Endocrine disruption and non-Hodgkin’s lymphoma	(Chen et al. 2018; Curi et al. 2019; Ju et al. 2019)
	Mecoprop	An auxin analog that stimulates uncontrollable and lethal growth in plants.	III	Skin and mucus membrane reactions. Nausea, diarrhea, delirium, bizarre, and muscular dystrophy	Immune system disruption, non-Hodgkin’s lymphoma, interference with blood clotting.	(Metayer et al. 2013)
Chloroacetanilide	Acetochlor	Long-chain fatty acid inhibitors	IV	Irritating to skin, eyes, and respiratory tract	No studies have shown its chronic health effect on man	(Yu et al. 2017; Tian et al. 2019; Chang et al. 2020)
Anilide	Propanil	Inhibition of photosystem II	III (oral) and IV (dermal)	Irritating to skin, eyes, and respiratory tract	No consensus on the chronic health impact	(Villarroel et al. 2003; Roberts et al. 2009)
Triazine	Atrazine	Photosystem II inhibitor	IV	Irritant to skin, eyes, respiratory tract. Abdominal cramp, nausea, and diarrhea	No consensus on the chronic impact	(Lim et al. 2009; Nwani et al. 2010; Jestadi et al. 2014; Gustafson et al. 2015; de Paiva et al. 2017)
Benzoic acid or chlorophenoxy herbicide	Dicamba	An auxin analog that stimulates uncontrollable and lethal growth in plants.	III	Skin allergy, anorexia, nausea, muscular dystrophies, bradycardia, and dyspnoea	Liver, intrahepatic bile duct cancer, lymphocytic leukemia, mantle cell lymphoma	(Soloneski et al. 2016; Lerro et al. 2020)
Organophosphorus	Glyphosate	Shikimic acid pathway inhibitors	III	Irritating to skin, eyes, and respiratory tract.	No consensus on the chronic health impact	(Myers et al. 2016; Soloneski et al. 2016; Tarazona et al. 2017; Van Bruggen et al. 2018; Peillex and Pelletier 2020)
Village	Paraquat	Photosystem 1 inhibitors	II	Chest burn, itches in the mouth, throat, chest, upper abdomen, vomiting, giddiness, migraine, lethargy, cracked and dry feet and palm, and skin ulceration.	Parkinson’s disease, kidney and heart failure, and pulmonary fibrosis	(Nakai et al. 1981; Dinis-Oliveira et al. 2008; Yu et al. 2014; Hashemi et al. 2017; Elenga et al. 2018; Isha et al. 2018)
Anilines	Pendimethalin	Inhibit cell division and elongation.	IV	Irritating to skin, eyes, and respiratory tract	No consensus on the chronic health impact	(Strandberg and Scott-Fordsmand 2004; Vighi et al. 2017; Ahmad et al. 2018)
Pyridinones	Fluoridone	Interfere with the carotenoid biosynthetic pathway	IV	Irritating to skin, eyes, and respiratory tract	No consensus on the chronic health impact	(Hamelink et al. 1986; Yi et al. 2011)

Apart from the exposure to toxic chemicals from herbicides and insecticides as a result of man’s occupational, accidental, or intentional activities, many individuals still come in constant with residues from everyday foods and beverages (Kumar et al. 2020). These chemical residues are recalcitrant and are difficult to wash off from agro-produces thoroughly. Hence, they are consumed as long as their concentration may be lower than the legislatively determined safe concentration. However, certain toxicity levels can still be experienced due to the synergistic effects of several minute residues that may be ascertained to be safe (or have a lower concentration than the legislatively acceptable dose) (Yigit and Velioglu 2020). Although synthetic insecticides and herbicides are classified into several groups based on their mode of action and the class of their active chemicals, their impact on health may vary among synthetic pesticides in the same class.

## Sustainable alternative to toxic synthetic pesticides—natural metabolites from *Zanthoxylum species*

Malaria, leishmaniasis, and other diseases whose causative organisms are transmitted by vectors are increasingly causing significant problems for humans, leading to many sicknesses and death (Eder et al. 2018). Moreover, agricultural activities for food production are threatened by disease-carrying pests and destructive insect pests (Chanda et al. 2017). This situation is aggravated by the growing resistance of these vectors to available insecticides and synthetic pesticides, making the vectors thrive and promoting the spreading of disease-causing organisms (Wilson et al., 2020). These synthetic chemicals also constitute acute and chronic toxicity to humans because of their poor selectivity. Moreover, their persistent nature fosters their accumulation in the environment, causing significant harm to living organisms in soil and water bodies (Nwani et al. 2013, 2021). Hence, the need for a more sustainable alternative for disease prevention, vector control, and agricultural purposes, with adverse effects on human health, our environment, and the ecosystem.

In recent years, research efforts have been directed into seeking and applying secondary metabolites or natural products from plants, microbes, or animals for sustainable management of human disease vectors and agricultural pests (Okeke et al. 2021; Ezeorba et al. 2022; Okagu et al. 2022; Enechi et al. 2022). These natural products are usually environmentally friendly and non-toxic to humans and effectively repel or kill these harmful pests. We shall review recent findings on interesting metabolites from *Zanthoxylum species* as a potential source of sustainable natural pesticides.

The *Zanthoxylum* genus is an exciting member of the Rutaceae family containing over 250 species of deciduous and evergreen trees, shrubs, and climbers, which are native to the subtropical regions of the world. Members of the *Zanthoxylum species* have reported interesting metabolites and volatile organic chemicals with tremendous pesticide activities. Although some studies have reported these phytochemicals of agricultural and pest-control interest to be present in polar fractions (ethanoic, methanolic, and aqueous fractions) of different *Zanthoxylum* plant parts (fruits, leaves, and bark), the majority of studies have identified these phytochemicals in the fractions of the essential oil obtained either by hydro or steam distillation. In more detail, we shall discuss some of these *Zanthoxylum* metabolites based on their application as (a) animals and human disease vector control, (b) plant foliage insect pest control, (c) stored grain pest control, (d) phytogenic fungi control, and (e) weed control.

### Zanthoxylum species for animals and human disease vector control

Vectors are carriers of disease pathogens for transmission to the host, majorly humans and animals (Wilson et al. 2017). Several studies have identified some *Zanthoxylum species*’ plant extracts, fractions, and metabolites with insecticidal, larvicidal, pupicidal, or repellency of animal and human disease vectors. Common examples of these vectors (Table 3) are *Aedes aegypti* and *Aedes albopictus* (mosquito variants that are a carrier of yellow fever, zika fever, dengue fever, and chikungunya viruses), Culex pipiens pallens (mosquito variants and carrier of bancroftian filariasis and West Nile virus), Anopheles gambiae (carrier of *Plasmodium falciparum*), and Stomoxys calcitrans (stable flies, vector of many blood-borne zoonotic diseases) (Wilson et al. 2020; Chala and Hamde 2021).

**Table 3:** Natural metabolites from *Zanthoxylum species* for animal and human diseases vector control

*Zanthoxylum* sp.	Plant part	Extract	Isolated and characterized compounds	Biological activities	Animal/human insect vectors	Ref.
*Zanthoxylum limonella*	Dried fruits	Essential oil	Limonene	Larvicidal and pupicidal (LC_50_ ranging 2.5–3.7%)	*Aedes aegypti* and *Aedes albopictus*	(Soonwera et al. 2022)
*Zanthoxylum limonella*	Dried fruits	Essential oil	N/A	Adulticidal (mortality in 24 h with LC_50_ of 5.7–6.0%, larvicidal (100% larva mortality in 12–24 h) LT50 for their larva and pupae is 0.24–4.11 h, and oviposition deterrents (negative oviposition index of -0.64 to -1.00	*Aedes aegypti* and *Culex quinquefasciatus*	(Soonwera and Phasomkusolsil 2017)
*Zanthoxylum piperitum*	Bark	Lignans and alkaloids	Lignin [(–)-asarinin, sesamin and (+)-xanthoxylol-γ,γ-dimethylallylether (XDA)] and alkaloid [pellitorine]	Insecticidal and larvicidal LD_50_ (XDA) 0.27 and 0.24 mg/l against C. pipiens pallens and Ae. Aegypti, respectively. Lower than synthetic temephos with LD50 of 0.006 and 0.009 mg/l	Wild *Culex pipiens pallens* and *Aedes aegypti*	(Kim and Ahn 2017)
*Zanthoxylum heitzii*	Bark, leaf, and seed	n-Hexane using accelerated solvent extraction and Soxhlet extraction	N/A	Anti-protozoan, anti-malaria parasite (LD_50_ = 102 ng/mg)	*Anopheles gambiae*	(Overgaard et al. 2014b)
*Zanthoxylum fagara* (*L.*)	Aerial parts	Methanol and ether extracts	N/A	Larvicidal LC_50_ of 338.8–396.2 μg/ml	Dengue mosquito	(De La Torre Rodriguez et al. 2013)
*Zanthoxylum piperitum* and *Zanthoxylum armatum*	Seed	Essential oil	Cuminaldehyde, thymol, (1S)-(-)-verbenone, (-)-myrtenal, carvacrol, (S)-(Z)-verbenol, *Zanthoxylum* piperitum steam distillate, cuminyl alcohol, *Zanthoxylum* armatum seed oil, piperitone, (-)-(Z)-myrtanol, and citronellal	Insecticidal fumigant toxicity- LC_50_, 0.075–0.456 μg/cm^3^ And ache inhibition (IC50—1.20–2.73 mM).	*Stomoxys calcitrans* (female stable fly)	(Hieu et al. 2012a)
*Zanthoxylum piperitum*	Fruit	Essential oils	N/A	Insecticidal repellent better than synthetic N,N-diethyl-3-methylbenzamide (DEET)—protection time of 1.5–2.5 h (EO) vs. 3.5–5.5 h (DEET). 100% bite protection by EO vs. 99.7% bite protection by DEET.	Female *Aedes aegypti*, *Aedes gardnerii*, *Anopheles barbirostris*, *Armigeres subalbatus*, *Culex tritaeniorhynchus*, *Culex gelidus*, *Culex vishnui* group, and *Mansonia uniformis*	(Kamsuk et al. 2007a)

A recent study by Soonwera et al. (2022) identified limonene as the major component of the essential oil of *Zanthoxylum limonella* dried fruits having larvicidal and pupicidal activities against Aedes aegypti and Aedes albopictus with an LC_50_ range of 2.5–3.7 % and LT_50_ range of 0.1–0.3 h). An earlier study from the same lab reported that the essential oil fraction of the *Z. limonella* dried fruits was potent against *Aedes aegypti* and *Culex quinquefasciatus*, expressing adulticidal, larvicidal, pupicidal, and oviposition deterrence activities (Soonwera and Phasomkusolsil 2017). In detail, well-developed adult insects expressed lethality (LC_50_ of 5.7–6%) after 24 h of exposure to 10% oil in ethanol. In contrast, a 100% mortality of larva and pupae was reported after the same time. Finally, it was discovered that the EO of *Z. limonella* caused a negative oviposition index (ranging from –0.89 to −1.00) in both insect vectors, implying a deterrence in their egg-producing activities and reproduction (Soonwera and Phasomkusolsil 2017).

*Zanthoxylum piperitum* is another exciting source of insecticidal and vector-controlling metabolites extensively explored in recent studies. Different studies have isolated valuable metabolites from the plant's barks, fruits, and seeds (Kamsuk et al. 2007a; Hieu et al. 2012a; Kim and Ahn 2017). Important and bioactive lignans and alkaloids extracted from the bark of *Z. piperitum* were reported with insecticidal and larvicidal activities against the wild Culex pipiens pallens and Aedes aegypti (Kim and Ahn 2017). The spectrometric analysis identified (–)-asarinin, sesamin, and (+)-xanthophyll-γ,γ-dimethylallylether (XDA) as the major lignins, while pellitorine was the most abundant alkaloid in the fraction. Purified XDA gave a reasonable LC_50_ of 0.27 and 0.24 mg/l against *C. pipiens pallens* and *Ae. Aegypti*, respectively, although lower than synthetic temephos (LD50 of 0.006 and 0.009 mg/l, respectively) (Kim and Ahn 2017). Older studies on the same plants (as summarized in Table 3) have reported the potencies of the essential oils extracted from their fruits and seeds as insect fumigant and repellent against the zoonotic vectors (Stomoxys calcitrans and Mansonia uniformis) and numerous insect vectors of human diseases (Kamsuk et al. 2007a; Hieu et al. 2012a).

In another study, essential oils from *Z. acanthopodium* aerial parts were shown to be cytotoxic to malaria vectors, *A. anthropophagus* and *A. Sinensis*, with LC_50_ and LC_90_ values of 36.00 and 101.49 mg/L and 49.02 and 125.18 mg/L, respectively, supporting its traditional use in killing insects in China and some other Asian countries (He et al. 2018). The authors further showed that estragole, eucalyptol, β-caryophyllene, *cis*-linalool oxide, and *cis*-limonene oxide were the major constituents of the essential oils, with estragole and eucalyptol suggested to be responsible for the cytotoxicity of the essential oils against the two vectors. Estragole was cytotoxic against *An. Anthropophagus* and *An. Sinensis* with LC_50_ and LC_90_ values of 38.56 and 95.90 mg/L and 41.67 and 107.89 mg/L against *An. Anthropophagus* and *An. Sinensis*, respectively. Similarly, eucalyptol was cytotoxic with LC_50_ and LC_90_ values of 42.41 and 114.45 mg/L and 45.49 and 124.95 mg/L against *An. Anthropophagus* and *An. Sinensis*, respectively.

Essential oil from *Z. bungeanum* fruits was shown to have repellant effects against the malaria vector (the malaria parasite-carrying insect), *Aedes aegypti*, supporting the traditional use of the plant in Thailand and other countries (Chaithong U, Kamsuk K, ChoochoteW, Jitpakdi A, Tippawangkosol P, Tuetun B, Champakaew D 2006). In addition, the repellency of the essential oil was compared with a synthetic repellant, N, N-diethyl-3-methyl benzamide, against selected mosquitoes; *A. Gardner*, *A. barbirostris*, *Armigeres subalbatus*, *C. tritaeniorhynchus*, *C. gelidus*, *C. vishnui group*, and *Mansonia uniformis*. The essential oil was shown to effectively repel all the insects (100% repellency) compared to the synthetic repellant, which repelled only 99.7% of the insects in the field; however, the synthetic insecticide had slightly higher repellant action in the laboratory (Kamsuk et al. 2007b). Further investigation into the mechanism of this repellency and the specific contents of the essential oil responsible for the activity is recommended. In another study, essential oil from *Z. armatum* seeds gave good cytotoxic activity when exposed to the larva of *Aedes aegypti*, *Culex quinquefasciatus*, and *Anopheles stephensi* with LC_50_ values of 49, 54, and 58 ppm*,* respectively, supporting the traditional use of the plant in vector control for malaria prevention in India (Tiwary et al. 2007). Further study on the insecticidal activities of *Z. armatum* showed that extracts of the stem bark strongly repelled *A. gambiae* (Mikolo et al. 2009), showing that the plant has promising applications in controlling *Anopheles species* and preventing malaria as well as minimizing the environmental concerns associated with synthetic insecticides that are used as vector control strategies.

Additionally, Samita et al. (Samita et al. 2013) prepared different solvent extracts of the stem bark of *Z. pyracantha*, a medicinal plant used in killing mosquitoes in Kenya, and tested their activities against the larva of *A. gambiae*. The extracts elicited larvicidal activities, with dichloromethane extract being the most active. Bioactivity-directed fractionation of the dichloromethane extract yielded zanthoxoaporphines A, B, C, and sesamin. These isolates also gave potent larva-killing activities with sesamin and zanthoxoaporphine A, showing excellent larvicidal potentials with LC_50_ values of 10.3 and 11.1 μg/ml after 72 h exposure. Similarly, Hieu et al. (Hieu et al. 2012b) evaluated the potential application of two *Z. species*, viz. *Z. bungeanum*, and *Z. armatum*, as natural insecticides. Solvent extracts and essential oils from the species and their secondary metabolites (cumin aldehyde, thymol, (1*S*)-(-)-verbenone, (-)-myrtenal, carvacrol, (*S*)-(*Z*)-verbenol, and cuminyl alcohol from *Z. bungeanum,* and piperitone, (-)-(*Z*)-myrtanol and citronellal from *Z. armatum*) demonstrated moderate toxicity against *Stomoxys calcitrans* (with LC_50_ values of 0.075–0.456 μg/ml). However, the insecticidal activities of the plants and their compounds were lower than those of two synthetic organophosphorus insecticides, chlorpyrifos, and dichlorvos. Some of the isolated compounds, citronellyl acetate, α-pinene, thymol, carvacrol, and α-terpineol, inhibited acetylcholinesterase activities in the insect. However, the acetylcholinesterase-inhibitory effect was not directly linked with the insecticidal activities.

The activities of *Z. heitzii* against many types of insects have been reported (Mikolo et al. 2009; Overgaard et al. 2014a), supporting the traditional application of the root bark decoctions in controlling vectors of many human diseases. Secondary metabolites isolated from *Z. heitzii* stem bark (dihydronitidine, caryophyllene oxide, pellitorine, and sesamin) showed insecticidal activities against the adult and larvae stages of *A. gambiae*. Specifically, pellitorine inhibited both stages (with LD_50_ values of 50 ng/mg and 13 μg/ml against insects and larvae, respectively), while caryophyllene oxide and sesamin inhibited only the larval stage with LD_50_ > 150 μg/ml. When combined, these compounds were also synergistic, exhibiting higher toxicity to both insect stages than their individual effects when used alone (Moussavi et al. 2015). This report has positioned phytochemicals from *Z. heitzii* stem bark, especially pellitorine, as natural insecticides that can target both adult and larvae stages of *A. gambiae*. It is worth mentioning that the above study has some limitations in not using standard insecticides to compare the activity of the herbal compounds. To strengthen the study’s designs, researchers working on biological activities of bioactive compounds, whether synthetic or natural products, are hereby recommended to ensure they include a group treated with standard drugs to make room for comparison of the efficacy of the test compound(s) with a reference standard(s).

Future studies should delve into the search for more potent metabolites from other *Zanthoxylum species* as well as other plants. Moreover, further characterization of these metabolites and their nanoformulations for their controlled release and delivery may improve their bioactivities (Okeke et al. 2022c).

### *Zanthoxylum species* and its metabolites for agricultural field insect pest control

Agricultural and food crop production is greatly affected by the infestation of field insect pests, which results in their overall decrease in viability and productivity. These insects are parasitic, feeding, and taking shelter from different parts of the plants for their survival, regardless of the detrimental harm they inflict on the plants. Metabolites of several *Zanthoxylum species* have been reported to play vital roles in controlling the field of insect pests to boost agricultural productivity and maintain the integrity of our environment and ecosystem (Table 4).

**Table 4:** *Zanthoxylum* metabolites and extract for controlling agricultural field insect pest

*Zanthoxylum sp.*	Plant part	Extract	Isolated and characterized compounds	Biological activities	Pest/insect (plant disease)	Possible mechanism of actions	Ref.
*Zanthoxylum rhoifolium*	Fruits	Essential oil—nanosphere prepared by nanoprecipitation	β-phellandrene (76.8%), β-myrcene (9.6%), and germacrene D (8.3%)	Insecticidal effects 84.3% vs. 64.8% mortality for nanoformulation and free, respectively, in 1.5% EO suspension	*Bemisia tabaci* (*Sternorrhyncha: Aleyrodidae*)—a bean plant pathogen	Reduction of insect oviposition by 71% Second instar nymphs mortality by ≥64%	(Pereira et al. 2022)
*Zanthoxylum armatum*	Fruits pericarp and leaves	N-hexane, methanol, ethyl acetate, aqueous extract		Insecticidal LC_50_ of 0.179 (n-hexane)—5.97% (aqueous) Compare to azadirachtin 0.15EC (LC_50_ of 0.239%) and a comparable lethal time (LT _50_)—60 hr	Oriental leaf worm, *Spodoptera litura* (*Fabricius*)	Contact and oral toxicity and sub-lethal effects (including antifeedant and ovicidal action)	(Kaleeswaran et al. 2018)
*Zanthoxylum bungeanum*	Fruits	Ethanol extract	Linalool and piperine	Insecticidal	*Nephotettix cincticeps* (female adult and third-instar nymphs of the rice pest, leafhopper)	Glutathione S tranferase (GST), carboxyl esterase (CarE), and acetylcholinesterase (AchE)	(Chakira et al. 2017)
*Zanthoxylum rhoifolium Lam.*	Leaf and branch	Extracts	N/A	Insecticidal	Workers of *Atta sexdens* L. cutting-ants	N/A	(Gomes et al. 2016)
*Zanthoxylum armatum*	Leaf	n-Hexane, ethanol, methanol, and chloroform extract	2-Undecanone (19.75%) and 2-tridecanone (11.76%)	Insecticidal and larvicidal activities with LC_50_ value of 2988.6–16750.6 ppm	The diamond back moth, *Plutella xylostella*		(Kumar et al. 2015)
*Zanthoxylum armatum*	Fruit	Essential oil	N/A	Insecticidal (LC_50_—55–60 ppm) after 48 h contact time)	*Aphis cracccivora*		(Tewary et al. 2005)

*Zanthoxylum rhoifolium* has been reported as a rich source of valuable metabolites for deterring and killing field pests. A recent study has shown that the free essential oil of *Z. rhoifolium* fruits, which contained majorly β-phellandrene (76.8%), β-myrcene (9.6%), and germacrene D (8.3%), had insecticidal activities (64.8 % mortality) against a bean plant pathogen (*Bemisia tabaci*) in 1.5% EO suspension (Pereira et al. 2022). Moreover, the nanoformulation of its essential oils to generate a homogenous nanosphere by nano-precipitation improved the insecticidal activities to about 84.3% mortality, 71% reduction in insect oviposition, and greater than 64% mortality of second instar nymphs (Pereira et al. 2022). Finally, nanoformulation improves the EO’s insecticidal activities, and the study showed improved photostability and stability in adverse conditions (Pereira et al. 2022). A previous study on the same plant, *Z. rhoifolium* (although with the extract of leaves and branches), has shown its great insecticidal activities against workers of Atta sexdens L. (cutting ants) (Gomes et al. 2016). The cutting ant is a well-known destructive plant pest that attacks the leaves and other aerial parts of several plants at different stages of growth (Mota Filho et al. 2021).

*Zanthoxylum armatum* is another species studied for its insecticidal activities against agricultural field pests. It was recently reported by (Kaleeswaran et al. 2018) that the n-hexane, methanol, ethyl acetate, and aqueous extract of *Z. armatum* fruit pericarp and leaves, though contact and oral toxicity, fostered the mortality of oriental leaf worms (*Spodoptera litura*), with an LC_50_ range of 0.179–5.97%. This insect pest majorly attacks and destroys the leaves of tobacco and cotton plants (Kaleeswaran et al. 2018). The insecticidal activities of the n-hexane fraction (LC_50_—0.179) were slightly better than a synthetic pesticide—azadirachtin, with LC_50_ of 0.239 %, although both with comparable lethal time (LT_50_—60 h). Moreover, the plant extracts also showed interesting antifeedant and ovicidal modes of action to curtail the spread of the field pests (Kaleeswaran et al. 2018). Another study adopted the use of spectrometry techniques to identify compounds present in the n-hexane extracts of *Z. armatum* leaves and discovered majorly two fatty acids - 2-undecanone (19.75%) and 2-tridecanone (11.76%), with impressive potencies against another field pest—diamondback moth (*Plutella xylostella*). In addition to the insecticidal activities, the extract also showed larvicidal activities with an LC_50_ of 2988.6–16750.6 ppm (Kumar et al. 2015). Finally, the essential oil of *Z. armatum* fruits was earlier reported to be potent against the field pest—*Aphis cracccivora*, when in contact with the pest for about 48 h, and LC_50_ between 55 and 60 ppm (Tewary et al. 2005)

Tringali et al. (2001) isolated sesamin, 1-hydroxy-3-methoxy-*N*-methylacridone, arborinine, xanthoxoline, 1-hydroxy-3-methoxyacridone, oblongine, tembetarine, magnoflorine, and hesperidin from ethanol extract of *Z. Clava-herculis* barks and examined their insecticidal activities against the larva of *Spodoptera littoralis* and *S. frugiperda*. It was reported that among these compounds, xanthoxoline showed the highest antifeedant activity by suppressing feeding patterns in *S. littoralis* and *S. frugiperda* larvae by 53% and 58%, respectively. This report positions xanthoxoline as a potential entity that demands further investigation as a larva-stage insecticide for *S. frugiperda* and *S. littoralis*. Other *Zanthoxylum species* with extracts or metabolites against field insect pests have been summarized in Table 4.

### *Zanthoxylum species* and their metabolites for the control of pests of agricultural stored products

To ensure an all-year-round supply of food crops and other agro products, there is a need to improve agro-storage systems to ensure that the viability and quality of agro-produce are maintained. Some insect pests specifically attack stored products fostering their spoilage (Kumar and Kalita 2017). *Zanthoxylum* metabolites in many studies have proven to be a sustainable alternative to synthetic pesticide to wage against insect pests of stored products. Moreover, these metabolites are non-toxic to humans even when consumed alongside stored food crops (Ke et al. 2019).

The dried stem bark and roots of *Zanthoxylum zanthoxyloides* were processed into fine powder to prevent infestation of *Callosobruchus maculatus*, a well-known insect pest that attacks Bambara nut, cowpea, and lentils. Ugwu et al. (2022) reported that 10 g of fine powder of *Z. zanthoxyloide* dried stem bark per 100 g of Bambara-nut caused about 80.67% mortality of *Callosobruchus maculatus* after about 120 h contact time. More so, an observable deterrence in oviposition and adult emergence was also reported as part of the mechanism of action of Z. *zanthoxyloide* against the pests (Ugwu et al. 2022). An earlier study obtained a similar result, which prepared and adapted the root powder of Z. *zanthoxyloide* to prevent the same insect pest—*C. maculatus*. Significant antifeedant activities were observed at a concentration of 5% (wt/wt) of the powder, and a 100% mortality was recorded after 3 days (Odeyemi et al. 2013).

Essential oils of *Zanthoxylum monophyllum* fresh fruits prepared by hydrodistillation have been reported in two studies to be potent against *Tribolium castaneum* (Red flour beetle) and *Sitophilus oryzae* (Prieto et al. 2011; Oviedo-Sarmiento et al. 2021). In the most recent study, Oviedo-Sarmiento et al. (2021), using GC-MS analysis, identified α-pinene (6.7%), β-pinene (35.3%), β-ocimeno (7.9%), and linalool (10.8%) from the essential oil fraction. The essential oil showed significant insecticidal and fumigant toxicity against the Red flour beetle, with an LC_50_ of 18.5 μL/L air. Moreover, the mechanism of insecticidal activities of the essential oil fraction owes to the fostering >50% inhibition of acetylcholinesterase (AChE), glutathione S-transferase (GST), and catalase (CAT) in the insect pest (Oviedo-Sarmiento et al. 2021). Contrarily, Prieto et al. (2011) previously reported the abundance of sabinene (25.71%), 1,8-cineole (9.19%), and cis-4-thujanol (9.19%) in the essential oil fractions of the same plant. Their studies showed the insecticidal and fumigant activities of the essential oil against *Sitophilus oryzae* (insect pests of stored rice products) with an EC_50_—222 μL L^-1^ air. Many factors may have contributed to the difference in phytochemical contents between the two studies, such as differences in analytical protocol, different plant growth conditions, and other salient processing (Prieto et al. 2011). In another study, the botanical fine powdered prepared by pulverizing dried fruits of *Zanthoxylum armatum Roxb* has also shown insecticidal activities against *Sitophilus oryzae L* (Rice weevils), causing about 70.67% inhibition at a concentration of 10 g/kg (Khanal et al. 2021).

Stored products destructive insects (*Tribolium castaneum*) have recently been deterred and killed by essential oil fraction of *Zanthoxylum limonella* seed and *Zanthoxylum planispinum* var. *dintanensis* leaves and fruit pericarps. Wanna and Satongrod (2020) reported that the essential oil fraction of *Z. limonella* seeds contained multiple phytochemicals with more abundance of (19.65%), 9-octadecanone (18.80%), and D-limonene (9.76%). About 10% concentration of the essential oil results in a 100% mortality of the insects’ eggs, larvae, and adults within 14 days,48 h, and 120 h, respectively. Conversely, the essential oil of *Z. planispinum* fruits and leaves had an abundance of oxygenated monoterpenes (linalool, sylvestrene, and terpinen-4-ol) from the fruits and 2-dodecanone from the leave extract. It was reported that purified 2-dodecanone showed interesting insecticidal activities against *T. castaneum* adults (LD_50_ = 2.54–23.41 μg/adults) after 2–4 hr contact post-exposure (Wang et al. 2019). Several other *Zanthoxylum species* have been processed for their metabolites against pests of stored agro-products, as summarized in Table 5.

**Table 5:** *Zanthoxylum* metabolites for pest control of agro products

*Zanthoxylum sp.*	Plant part	Extract	Isolated and characterized compounds	Biological activities	Pest/insect (plant disease)	Possible mechanism of actions	Ref.
*Zanthoxylum zanthoxyloides*	Dried stem bark	Fine powder with an electric blender	N/A	Insecticidal 10 g/100 g of BG powder concentration caused 80.67% mortality of adults after 120 h treatment time. Reduction in weevil perforation index to <50	*Callosobruchus maculatus* (Bambara groundnut disease)	Prevented oviposition and adult emergence Comparable to synthetic pesticide—2% actellic, resulting in 100% mortality after 24 h	(Ugwu et al. 2022)
*Zanthoxylum monophyllum*	Fresh fruits	Essential oils	α-Pinene (6.7%), β-pinene (35.3%), β-ocimeno (7.9%), and linalool (10.8%)	Insecticidal fumigant toxicity (LC_50_ of 18.5 μL/L air)	*Tribolium castaneum* (red flour beetle)	>50% inhibition of acetylcholinesterase (AChE), glutathione S-transferase (GST), and catalase (CAT)	(Oviedo-Sarmiento et al. 2021)
*Zanthoxylum armatum Roxb*	Fruits	Botanical fine powder	N/A	Insecticidal (70.67% inhibition at a concentration of 10 g/kg)	*Sitophilus oryzae L. Coleoptera*: *Curculionidae* Rice weevil		(Khanal et al. 2021)
*Zanthoxylum limonella*	Seeds	Essential oils	Multiple component with (19.65%), 9-octadecanone (18.80%), D-limonene (9.76%) with highest abundance	Insecticidal activity 10% conc of EO caused 100% mortality of insect eggs after 14 days, 120 h for adults, and 48 h for larvae	*Tribolium castaneum* (destructive insect of stored grain)		(Wanna and Satongrod 2020)
*Zanthoxylum planispinum var. dintanensis*	Leaves and fruit pericarp	Essential oil	Oxygenated monoterpenes (linalool, sylvestrene, and terpinen-4-ol) and 2-dodecanone from the leave extract	Insecticidal, 2-dodecanone (LD_50_ = 2.54 μg/adults—23.41 μg/adults) after 2–4 hr contact post-exposure.	Stored products insects (*Tribolium castaneum*, *Lasioderma serricorne*, and *Liposcelis bostrychophila* adults)		(Wang et al. 2019)
*Zanthoxylum zanthoxyloides*	Plant roots	Plant powder	N/A	Insecticidal and antifeedant with a mortality rate of 100% at 5% (wt/wt) conc for three days	*Callosobruchus maculatus*	Post-locomotion, oviposition, feeding behavior, developmental, and physiological processes	(Odeyemi et al. 2013)
*Zanthoxylum monophyllum*	Fruits	Essential oils	Sabinene (25.71%), 1,8-cineole (9.19%), and cis-4-thujanol (9.19%)	Insecticidal/fumigant (EC50 -222 μL L^-1^ air)	*Sitophilus oryzae*		(Prieto et al. 2011)
*Z. fagara*	Fruits	Essential oils	Germacrene D-4-ol (21.1%), elemol (8.35%), and α-cadinol (8.22%)	Insecticidal/fumigant EC_50_ 153.9 μL L^-1^ air	*Colletotrichum acutatum Simmonds*		(Prieto et al. 2011)
*Zanthoxylum usambarense*	Roots and bark	Dichloromethane	Canthin-6-one (fungicide) and pellitorine (insecticide), oxychelerythrine, norchelerythrine, (+)-sesamin, and (+)-piperitol-3,3-dimethylallyl ether	-	-	-	(He et al. 2002)

### *Zanthoxylum species* and their metabolites for controlling phytopathogenic fungi

Several disease-causing fungi and bacteria tremendously affect agriculture crops and animal production, leading to the loss of their quality and quantity and even death. Synthetic fungicides and bactericides have been popularized due to their mode of quick response to salvage an agro-business (Fausto et al. 2019). However, their impact on human and the environment is enormous and should not be overlooked. The use of natural products such as phyto-bio fungicides is still considered a more sustainable alternative (Zubrod et al. 2019). Recent studies have reported the application of some metabolites and volatile organic chemicals from *Zanthoxylum* as potent against phytopathogenic fungi and even bacteria (Table 6). Li et al. (2022b) recently reported the isolation of a plant’s volatile organic chemicals (Linalool) from the fruit pericarp of *Zanthoxylum schinifolium*. This PVOC caused membrane disruption to *Aspergillus flavus* (a post-harvest spoilage organism) when applied as a biofumigant with a MIC and MFC of 0.571 μL/mL and 0.857 μL/mL, respectively (Li et al. 2022b).

**Table 6:** *Zanthoxylum* metabolites as natural fungicides and bactericides

*Zanthoxylum sp.*	Plant part	Extract	Isolated and characterized compounds	Biological activities	Pest/insect (plant disease)	Possible mechanism of actions	Ref.
*Zanthoxylum schinifolium*	Pericarp	PVOCs	Linalool	Anti-phytogenic fungi biofumigants (MIC- 0.571 μL/mL MFC- 0.857 μL/mL)	*Aspergillus flavus* (post-harvest spoilage grain)	Membrane disruption	(Li et al. 2022b)
*Zanthoxylum bungeanum*	Pericarp	Methanolic extract	Flavonoids (quercetin, epicatechin, kaempferol-3-O-rhamnoside, and hyperoside)	Anti-phytogenic fungi (inhibited fungal growth by 48.5% and DON mycotoxin production (73.0%) DNA reduction by 85.5%	*Fusarium graminearum* (Fusarium head blight of wheat by deoxynivalenol mycotoxin production)	N/A	(Abbas and Yli-Mattila 2022)
*Zanthoxylum alatum*	Leaves	Essential oil and methanolic extract	Linalool (30.58%), 2-decanone (20.85%), β-fenchol (9.43%), 2-tridecanone (8.86%), β-phellandrene (5.99%), sabinene (4.82%), and α-pinene (4.11%)	Antifungal and antibacterial (pesticidal)	Fungi (*Alternaria alternata*, *Alternaria brassicae*, and *Curvularia lunata*) and bacteria (*Bacillus subtilis*, *Micrococcus luteus*, *Staphylococcus aureus*, and *Escherichia coli*).		(Guleria et al. 2013)
*Z. rhoifolium*	Fruits	Essential oils	β-Myrcene (59.03%), β-phellandrene (21.47%), and germacrene D (9.28%)	Antifungal fumigant (EC_50_ 140.1 μL L^-1^ air)	*Fusarium oxysporum*		(Prieto et al. 2011)`

*Fusarium graminearum* is a devastating fungi-pathogen of cereals and one of the causative agents for the Fusarium head blight disease of wheat. The organism also produces mycotoxin – deoxynivalenol, which fosters the progression of the disease in plants (Abbas and Yli-Mattila 2022). Recently, the flavonoid-rich methanolic extract of *Zanthoxylum bungeanum* fruit pericarp (containing quercetin, epicatechin, kaempferol-3-O-rhamnoside, and hyperoside majorly) could help combat the progression and spread of Fusarium head blight disease caused by *Fusarium graminearum* (Abbas and Yli-Mattila 2022). The extract (100 μg/mL) caused a decrease in *F. graminearum* growth by 48.5%, its DNA level by 85.5%, and its mycotoxin production by 73.0% in an in vitro bioassay (Abbas and Yli-Mattila 2022). Similarly, according to Prieto et al. (2011), the essential oil of *Z. rhoifolium* fruits, with β-myrcene (59.03%), β-phellandrene (21.47%), and germacrene D (9.28%) as major components, was effective as a fumigant against Fusarium oxysporum at an EC_50_ of 140.1 μL L^-1^ air.

Another study has reported the potential of the essential oil and methanolic extracts of *Zanthoxylum alatum* leaves as a fungicidal and bactericidal agent (Guleria et al. 2013). It was shown that several fungi (such as *Alternaria alternata*, *Alternaria brassicae*, and *Curvularia lunata*), as well as a few bacteria (*Bacillus subtilis*, *Micrococcus luteus*, *Staphylococcus aureus*, and *Escherichia coli*), were significantly inhibited or killed by the extracts with the major components of linalool (30.58%), 2-decanone (20.85%), β-fenchol (9.43%), 2-tridecanone (8.86%), β-phellandrene (5.99%), sabinene (4.82%), and α-pinene (4.11%) (Guleria et al. 2013). It is worth mentioning that several other studies have discovered the antibacterial and antifungal potential of some other natural products from *Zanthoxylum*, although not specific to plant pathogens, but rather as an alternative to antibiotics (Okagu et al. 2021a, b). Therefore, the need for future studies to put in a more concerted effort toward the discoveries, application, and scale-up of novel natural products from *Zanthoxylum* and other plants as alternatives to synthetic pesticides.

### *Zanthoxylum species* as a potential source of natural herbicides

The possible application of *Z. bungeanum* fruits as herbicides has been evaluated. Volatile compounds, especially eucarvone from the plant’s fruits, moderately inhibited the hypocotyl growth of lettuce seedlings (Sunohara et al. 2014). Similarly, extracts of *Z. bungeanum* leaves have been shown to suppress the germination and viability of *Medicago sativa*, *Lactuca sativa*, and *Raphanus sativa* by up to 80% (Li et al. 2009). Furthermore, xanthoxyline isolated from *Z. limonella* fruits was reported to drastically suppress the germination of *Amaranthus tricolor* and *Echinochloa crusgalli* (Charoenying et al. 2010). In addition, extracts of *Z. schinifolium* leaves and stems were reported to significantly halt the germination and seedling of *Triticum sativum* (Wu et al. 2012)*.*

To further exploit *Z. species* as a source of herbicidal compounds, Rios et al. (Rios et al. 2018) prepared different solvent extracts of *Z. fagara* that showed varied herbicidal activities against *Lactuca sativa* and *Lolium perenne*. Additionally, linarin, lupenone, tocopherol, and affineine were isolated from the most active extract. Only linarin showed a good phytotoxic effect by inhibiting energy production and respiration. These reports demonstrate that *Z. species* are potential sources of natural herbicides. Hence, further studies are recommended on screening other species and testing their chemical constituents against many known weeds. The herbicidal potentials of *Z. species* are summarized in Table 7.

**Table 7:** Summary of herbicidal effects of *Zanthoxylum species*

Plant species and parts used	Test substance	Results	References
*Z. bungeanum* fruits	Eucarvone isolated from it	Moderately inhibited hypocotyl growth of lettuce seedlings	(Sunohara et al. 2014)
*Z. bungeanum* leaves	Crude extract	Suppressed the germination and viability of *Medicago sativa*, *Lactuca sativa*, and *Raphanus sativa* up to 80%	(Li et al. 2009)
*Z. limonella* fruits	Xanthoxyline isolated from the fruit	Drastically suppressed the germination of *Amaranthus tricolor* and *Echinochloa crusgalli*	(Charoenying et al., 2010)
*Z. schinifolium* leaves and stems	Crude extract	Significantly stopped the germination and seedling of *Triticum sativum*	(Wu et al. 2012)
*Z. fagara* aerial parts	Multiple solvent extracts	Herbicidal activities against *Lactuca sativa* and *Lolium perenne*	(Rios et al. 2018)

## Limitations, future research directions, and conclusion

There is an urgent need to minimize the negative impact of human activities on the environment and prevent the health outcome associated with it. Part of these strategies is to search for alternative chemicals for controlling vectors and insects of agricultural importance, especially from natural products that are less toxic to humans and animals and eco-friendly. Scientists are exploiting the understanding that plants synthesize some chemicals to repel and kill insecticides that cause plant diseases to evaluate the potential applications of extracts of these plants and chemicals isolated from them as a source of natural insecticides and herbicides. In the several studies reviewed, extracts of different parts of *Z. species* and compounds isolated from them were shown to have promising insecticidal activities to several insect vectors, especially malaria parasite vectors.

Although the insecticidal activities of the plant extracts and chemicals derived from them were lower than the synthetic insecticides, efforts to minimize the environmental and health concerns of using synthetic insecticides underscore the need for further development of these plant secondary metabolites as natural insecticides. Similarly, crude extracts of *Z. species* and compounds isolated from them were shown to have potential application as natural herbicides by strongly inhibiting the growth and development of several weeds. Despite these exciting reports, the mechanisms of insecticidal and herbicidal activities recorded by some of the *Z. species* and their chemical constituents need to be investigated. This will position them as potential sources of natural insecticides and herbicides.

In addition, some studies failed to compare the insecticidal and herbicidal activities of *Z. species-*derived entities with synthetic insecticides and herbicides. Future research should be designed to compare the potency of natural compounds under evaluation for insecticidal and herbicidal activities and known insecticides and herbicides to provide a sound basis for making an informed decision on the benefits of plant-based insecticides/herbicides over their synthetic counterparts. Moreover, the advent of nanotechnology development has opened new avenues for the development of control release and precise targeting of these bioactive components to the insect and pests for better efficient results. In conclusion, there is a need for the transition from bench-to-market (i.e., real-life application) of research finding about natural products to lessen the impact of synthetic pesticide toxicities and promote healthier alternatives.

## Data Availability

All data generated or analyzed during this study are included in this published article.
